# Perioperative shingles diagnosis does not influence prosthetic joint infection rates after shoulder arthroplasty

**DOI:** 10.1007/s00590-026-04954-0

**Published:** 2026-09-09

**Authors:** Erik R. Nakken, Alex Mamonov, Daniel G. Whitney, William R. Aibinder

**Affiliations:** University of Michigan–Ann Arbor, Ann Arbor, USA

**Keywords:** Shoulder arthroplasty, Shingles, Herpes zoster, Outcomes, Infection, PJI

## Abstract

**Purpose:**

Shoulder arthroplasty (SA) utilization has increased substantially, with a corresponding rise in revision procedures, many driven by periprosthetic joint infection (PJI). Herpes zoster (HZ), or shingles, is increasingly prevalent among older and immunocompromised patients and shares overlapping risk factors with PJI. However, the association between shingles and postoperative complications following SA is unknown. This study evaluated the relationship between peri-operative shingles and postoperative complications after SA.

**Methods:**

Patient-level claims from adults ≥ 18 years undergoing SA between 2016 and 2022 from a 20% Medicare fee-for-service sample and the IBM^®^ MarketScan^®^ Commercial Database were retrospectively collected. Shingles diagnoses from one year preoperatively through one year postoperatively were identified. For postoperative shingles, the diagnosis date was required to precede the earliest censoring event (outcome occurrence, loss to follow-up, end of follow-up) to maintain appropriate temporality of events. Outcomes within one year included revision arthroplasty, irrigation and debridement (I&D), implant removal, or shoulder PJI. Incidence rates (IRs) per 100 person-years and incidence rate ratios (IRRs) were calculated for a composite primary outcome. Associations between shingles and outcomes were evaluated using three Cox regression approaches with covariate adjustment.

**Results:**

Among 26,543 patients, 1.4% had shingles preoperatively and 1.0% developed shingles on or after surgery. The IR of the primary outcome was lower in patients with (IR 3.78; 95% CI 2.20–5.36) than without shingles (IR 5.06; 95% CI 4.77–5.35), but without statistical significance (IRR 0.75; 95% CI 0.49–1.14). No analytic approach showed a significant association, and no effect modification was observed.

**Conclusions:**

Shingles diagnosed within one year before or after shoulder arthroplasty was not associated with increased risk of revision surgery, I&D, implant removal, or PJI within one year postoperatively.

## Introduction

Shoulder arthroplasty is a common orthopedic procedure with growing indications [1]. Shoulder arthroplasty utilization has grown with a reported 103.7% increase in primary shoulder arthroplasties performed per year between 2011 and 2017 [2]. Increased volume of shoulder arthroplasty cases reasonably correlates with an increased rate of revision surgery [3, 4]. Between 2005 and 2017, Dillon et al. cited a 282% increase in the number of shoulder arthroplasty cases performed at their institution, along with a 362% increase in the number of revision cases happening in the same time period [4]. Revisions may occur for a number of reasons, including instability, periprosthetic joint infection, component loosening, or soft-tissue deficiency [5]. Periprosthetic joint infections (PJI) contribute significantly to the amount of revisions performed, with infection rates from a sample of 82,498 patients yielding an infection rate of 0.98% [6]. This raises the question of contributing factors that may lead to increased rates of PJI in patients as shoulder arthroplasty case volumes continue to grow.

Herpes zoster (HZ) infection, commonly referred to as shingles, results from reactivation of varicella-zoster virus following characteristic aging or immunosuppression [7]. It presents as a painful neurological condition affecting more than one million people in the United States per year [7, 8]. The rate of individuals with shingles has been increasing in recent years, with older patients being at the highest risk [7, 9]. Symptoms include skin rash, postherpetic neuralgia, vision loss from ocular inflammation, vertigo, mobility impairment, meningoencephalitis, secondary bacterial infections, and facial paresis [7, 8, 10–13]. There is clear evidence that immunocompromised individuals remain at the highest risk for reactivation of varicella-zoster virus, leading to the onset of HZ [7]. Furthermore, immunosuppressive conditions including rheumatoid arthritis and diabetes are also associated with an increased risk of PJI [7, 12, 14]. 

Liao et al. note that risk factors for shingles, including immunosuppression and advanced age, may overlap with those for PJI after total knee arthroplasty [12]. Meta-analyses from Zhu et al. and Kunutsor et al. mention that immunosuppressive conditions may lead to a higher risk of PJI following total joint arthroplasty [14, 15]. No current literature exists analyzing the relationship between shingles and its effect on PJI rates in shoulder arthroplasty. The objective of this study was to assess the association between pre-surgical and post-surgical development of shingles on post-operative complications following shoulder arthroplasty. The hypothesis was that shingles would not be strongly associated with complications, including PJI, following shoulder arthroplasty.

## Methods

### Data source

This retrospective cohort study accessed administrative claims data from January 1, 2016 through December 31, 2022 obtained from two separate databases: a 20% random sample of Medicare fee-for-service beneficiaries (Parts A and B) and the IBM^®^ Watson Health MarketScan^®^ Commercial Research Databases. These databases were combined to capture a large, heterogeneous adult population spanning the full adult age spectrum, as the Commercial database represents primarily non-elderly (< 65 years) working individuals while the Medicare database represents elderly (≥ 65 years) individuals [16]. Combining these cohorts enhanced geographic diversity, representation, and generalizability.

Medicare is a federal insurance program for elderly individuals, and certain non-elderly individuals with disabilities or end-stage renal disease. While some beneficiaries are dually eligible for Medicaid, Medicare is the primary payer for covered services relevant to this study. The MarketScan Commercial Database contains de-identified claims from approximately 350 U.S. payers and includes insured employees, Consolidated Omnibus Budget Reconciliation Act continuants, and non-Medicare-eligible dependents enrolled in employer-sponsored plans. All data were de-identified prior to researcher access; therefore, informed consent was not required, and the Institutional Review Board determined the study to be exempt and “Not Regulated.”

## Cohort

Individuals were included if they underwent shoulder arthroplasty between January 1, 2017, and December 31, 2021, identified using Current Procedural Terminology (CPT) codes 23,470 or 23,472. Additional inclusion criteria were age ≥ 18 years at surgery, at least one year of continuous health plan enrollment prior to the index surgery (baseline period), and at least one day of continuous enrollment following surgery. The post-index ≥ 1-day enrollment requirement was selected to optimize representation and reduce selection bias, as the analytic methods allowed for right censoring due to loss of coverage or other reasons. Among 30,658 individuals who underwent shoulder arthroplasty during the study period (15,720 Medicare; 14,938 Commercial), 26,543 met all inclusion criteria and were included in the analysis.

## Exposure

The exposure of interest was herpes zoster (shingles). For each individual, the first diagnostic evidence of shingles was identified from the 1-year preoperative baseline period through the 1-year postoperative follow-up period using International Classification of Diseases, Tenth Revision (ICD-10) code B02.x. The cohort was stratified into those with no evidence of shingles and those with evidence of shingles during the baseline or follow-up period.

For individuals with shingles, the timing of the first shingles claim was recorded in days relative to the date of surgery, with negative values representing preoperative exposure (range − 365 to − 1 days) and positive values representing exposure on or after surgery (range 0 to 365 days). For individuals whose first shingles diagnosis occurred on or after surgery, the diagnosis date was required to precede the earliest censoring event, including outcome occurrence, loss to follow-up, or completion of the 1-year follow-up period.

## Outcomes

Outcomes were selected based on clinical relevance to shoulder arthroplasty and reliable identification in claims data. Four outcomes were assessed from the date of surgery through one year postoperatively: (1) revision shoulder arthroplasty (CPT 23473 or 23474), (2) irrigation and debridement (CPT 23030, 23031, 23035, or 23040), (3) shoulder arthroplasty removal (CPT 23334 or 23335), and (4) shoulder periprosthetic joint infection (PJI) identified using ICD-10 codes T84.50XA, T84.50XD, T84.50XS, T84.59XA, T84.59XD, or T84.59XS.

The primary outcome was a composite of all four events. Secondary outcomes examined each individual outcome separately when event counts permitted.

## Descriptive characteristics

Baseline characteristics included year of surgery, age at surgery, sex, and U.S. region of residence. Age was calculated precisely in the Medicare database using full birth date, whereas only birth year was available in the Commercial database, resulting in less precise age estimation. Insurance type (Medicare versus Commercial) was included for all individuals. For Medicare beneficiaries, original reason for Medicare entitlement and dual eligibility with Medicaid were also assessed.

Overall health status was measured using the Elixhauser Comorbidity Index (ECI), calculated during the 1-year baseline period as the sum of 30 comorbid conditions (score range 0–30) [17]. Race and ethnicity were not available in the Commercial database and, therefore, were not analyzed.

### Statistical analysis

Baseline characteristics were summarized for the full cohort and stratified by shingles exposure status. Incidence rates (IRs) with 95% confidence intervals (CIs) for the primary outcome were calculated per 100 person-years for the full cohort and sub-cohorts, and crude incidence rate ratios (IRRs) compared individuals with and without shingles. Cumulative incidence of the primary outcome was visualized using the Fine-Gray approach [18]. Individuals were followed until outcome occurrence, loss to follow-up, or completion of the 1-year follow-up period, whichever occurred first.

To evaluate the clinical impact of shingles on postoperative risk, three Cox proportional hazards modeling approaches were used, each analyzed with unadjusted and adjusted models. The first approach treated shingles as a binary baseline exposure (yes/no) to assess whether any preoperative shingles exposure was associated with postoperative complications. The second approach examined the timing of preoperative shingles exposure by modeling shingles as a baseline exposure by quarter, from quarter four(− 365 to − 274 days) through quarter one(− 91 to − 1 days), compared with no baseline exposure. The third approach treated shingles as a time-varying exposure from the baseline period through postoperative follow-up. Individuals with baseline shingles or shingles on the day of surgery were considered exposed from day zero, whereas individuals who developed shingles postoperatively contributed unexposed time until diagnosis and exposed time thereafter.

Effect size was reported as hazard ratios (HRs) with 95% CIs. Due to limited outcome events in the shingles-exposed group, Model one adjusted for age, sex, and ECI. To assess robustness to residual confounding, Model two additionally adjusted for insurance type and year of surgery. Sensitivity analyses focused on qualitative comparison of Model one and Model two estimates. Effect modification by covariates was tested, and proportional hazards assumptions were evaluated using weighted Schoenfeld residuals.

As part of the Data Use Agreement, estimates based on one to ten cases were suppressed for de-identification purposes. Analyses were performed using SAS version 9.4 (Cary, NC), and statistical significance was defined as a two-sided *P* value < 0.05.

## Results

### Descriptive characteristics

Of the full cohort of adults that underwent shoulder arthroplasty (*n* = 26,543), 1.4% (*n* = 358) had their first evidence of shingles during the 1-year baseline period while another 1.0% (*n* = 271) had their first evidence on the day of or after surgery, but prior to any other censoring event. Descriptive characteristics for the full cohort (*n* = 26,543) and the sub-cohorts with (*n* = 629) and without (*n* = 25,914) shingles in the baseline or follow-up period are presented in Table 1. Notably, the sub-cohort with versus without shingles was on average 3.0 years older, had a higher proportion of women (65.0% versus 51.1%), was more likely to be covered by Medicare insurance (69.0% versus 55.1%), and had a slightly higher Elixhauser Comorbidity Index score (median score of four versus three).

### Incidence and risk of the primary and secondary outcomes

**Fig. 1:**
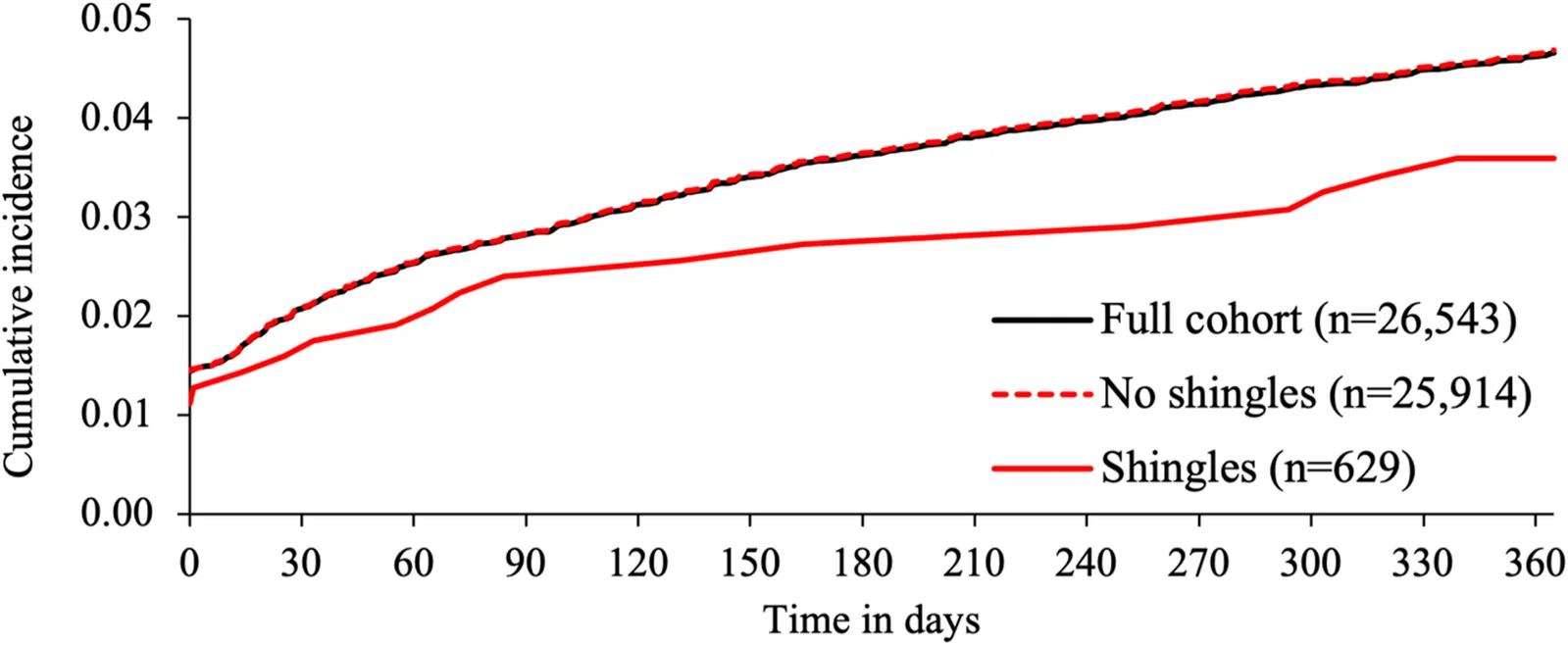
Cumulative incidence plot of the primary outcome (revision surgery, I&D, implant removal, or PJI) following shoulder arthroplasty for the full cohort and based on diagnostic evidence of shingles

The censor reason, IR, and IRR of the primary outcome and the risk of the secondary outcomes are presented in Table 2 and the cumulative incidence plot of the primary outcome is presented in Fig. 1. Notably, loss to follow-up was lower for the sub-cohort with versus without shingles (10.2% versus 15.3%). The IR (95% CI) per 100 person-years of the primary outcome (composite of all four outcomes) was 3.78 (2.20–5.36) for the sub-cohort with shingles in the baseline or follow-up period and 5.03 (4.75–5.32) for the sub-cohort without shingles; the IRR (95% CI) was 0.75 (0.49–1.14). As there were too few outcome events for the sub-cohort with shingles for most of the secondary outcomes, the IR and IRR were not estimated and only the risk estimate was presented.

### HR of the primary outcome

The HR of all models across all three analytic approaches are presented in Table 3. Regardless of how the shingles exposure was treated, the model findings suggest no strong association between shingles and the primary outcome before or after adjusting for covariates. Using Model 1, there was no strong evidence of effect modification between shingles and age, sex, or the Elixhauser Comorbidity Index, and using Model 2, there was no strong evidence of effect modification by insurance type or year of surgery in any of the three analytic approaches (*P* for interaction, all > 0.05). Finally, there was no evidence that the proportional hazards assumption was violated in any approach (*P* ranged from 0.499 to 0.722).

## Discussion

Using nationwide real-world clinical data from large, heterogeneous cohorts, this study found that shingles, from 1-year pre-operative up to 1-year post-operative, was not associated with complications following shoulder arthroplasty among Medicare and Commercial insurance beneficiaries. This finding was consistent across three different analytic approaches that provide complementary clinical insights, from pre-surgical risk stratification to post-surgical healthcare management. Moreover, the lack of effect modification suggests that the little-to-no effect of shingles on post-operative complications was relatively consistent across age, sex, Elixhauser Comorbidity Index scores, insurance type, and year of surgery strata. Although the point estimate suggested a lower incidence of complications among patients with shingles, this likely reflects residual confounding and healthcare utilization bias rather than a protective effect, and no clinically meaningful association was observed across multiple analytic approaches.

The incidence of shoulder arthroplasty, and, therefore, its associated complications, continue to rise [2–4]. Although shingles may arise from an immunocompromised state and is a reactivation of a prior infection [7], this has not been found to be linked to an increased risk in prosthetic joint infection within one year from surgery in our study. If we had discovered an association, it would be difficult to firmly conclude if the herpes zoster virus reactivation itself or the immunocompromised state of the patient is the primary etiology of PJI, as shingles could serve as a surrogate of immunocompromise. However, neither of these variables have been found to increase PJI risk in the current study.

This study has several imitations. Claims data does not contain a running medical history, and therefore, medical conditions can only be identified if there was a reimbursement claim containing a pertinent code during the study period. ECI was utilized as a marker for overall health status during adjustment in the statistical analysis. However, additional health status variables such as corticosteroid use, chemotherapy, smoking status, obesity, nutritional status, etc. are unavailable in claims data and there may be unmeasured clinical severity that we could not account for, thus introducing confounding bias. Important diagnostic information, such as the severity of shingles, treatment and cessation of shingles, and chronicity of shingles cannot be accurately measured. Specifically, due to the nature of the study we are unable to assess whether there is a difference in PJI risk based on active lesions and lesions located on the operative side or near the operative site. Nevertheless, the lack of this diagnostic information does not necessarily bias this study; rather, it prevents a more nuanced understanding of variation in risks based on clinical features of the shingles diagnosis, which future research will be needed to address. This study was designed to address the question via the Cox modelling approaches. While the temporality of postoperative shingles with other censoring events (e.g., lost to follow-up, end of follow-up, outcome occurrence) was appropriately accounted for in these models, the crude descriptive analysis of the outcome did not account for this temporality. As a result, there may be interpretive ambiguity in the crude analyses in regard to timing of events for the subgroup that developed shingles postoperatively. Thus, interpretations from this study are better suited if derived from the Cox modelling approaches as opposed to the crude description of the outcome. However, we do recognize that this lack of detail of the singles infection may bias findings towards the null. Our small shingles cohort (*n* = 629) and secondary outcomes with suppressed counts due to low event numbers (Table 2), are a limitation. We acknowledge the possibility of type II error, and that the absence of statistical significance does not necessarily exclude a potential modest increase in risk after shingles diagnosis. Lastly, the first shingles vaccine was introduced in 2006, and we are unable to assess the vaccination status of our included patients [19]. 

In this large, multi-database retrospective cohort study of patients undergoing shoulder arthroplasty, a diagnosis of herpes zoster in the year before or after surgery was not associated with an increased risk of revision surgery, irrigation and debridement, implant removal, or prosthetic joint infection within one year postoperatively. These findings were consistent across multiple analytic approaches and robust to adjustment for demographic and comorbidity burden, with no evidence of clinically meaningful effect modification. Although herpes zoster shares several risk factors with periprosthetic joint infection and other postoperative complications, our results suggest that its occurrence alone should not be considered an independent risk factor for early failure or infectious complications following shoulder arthroplasty. This study provides evidence for surgeons to guide patients through the perioperative decision-making process. Surgeons should use these data and their best clinical judgement to determine safety with proceeding with arthroplasty in patients with a shingles diagnosis. There is a need for future research to evaluate whether disease severity, antiviral treatment, or immunization status may influence longer-term outcomes.

**Table 1:** Descriptive characteristics for the full cohort and based on diagnostic evidence of shingles among adults undergoing shoulder replacement

	Full cohort (*n* = 26,543)	No shingles cohort (*n* = 25,914)	Baseline or follow-up shingles cohort (*n* = 629)
*Age in years*			
Mean (SD)	66.7 (10.0)	66.7 (10.0)	69.7 (9.5)
Median (IQR)	66.5 (60.0, 73.9)	66.3 (60.0, 73.9)	70.4 (63.0, 76.5)
*Sex, % (n)*			
Men	48.6 (12,903)	48.9 (12,683)	35.0 (220)
Women	51.4 (13,640)	51.1 (13,231)	65.0 (409)
*U.S. region of residence, % (n)*			
Northeast	12.7 (3,373)	12.7 (3,281)	14.6 (92)
Midwest	25.5 (6,755)	25.5 (6,606)	23.7 (149)
South	43.1 (11,439)	43.1 (11,160)	44.4 (279)
West	18.8 (4,976)	18.8 (4,867)	17.3 (109)
*Insurance type, % (n)*			
Medicare	55.4 (14,707)	55.1 (14,273)	69.0 (434)
Commercial	44.6 (11,836)	44.9 (11,641)	31.0 (195)
Original reason for Medicare entitlement, % (n)*			
Old age and survivor’s insurance	81.5 (11,985)	81.5 (11,630)	81.8 (355)
Disability insurance benefits (DIB)	18.3 (2,689)	18.3 (2,613)	17.5 (76)
End-stage renal disease (ESRD)	0.2 (22)	**	**
Both DIB and ESRD	0.1 (11)	**	**
Medicare-Medicaid dual eligibility, % (n)*	7.8 (1,146)	7.8 (1,110)	8.3 (36)
*Elixhauser Comorbidity Index*			
Mean (SD)	3.6 (2.7)	3.6 (2.7)	4.2 (3.0)
Median (IQR)	3 (2, 5)	3 (2, 5)	4 (2, 5)
*Year of index surgery, % (n)*			
2017	12.7 (3,368)	12.7 (3,284)	13.4 (84)
2018	13.2 (3,510)	13.2 (3,425)	13.5 (85)
2019	14.6 (3,887)	14.7 (3,804)	13.2 (83)
2020	15.1 (4,001)	15.2 (3,935)	10.5 (66)
2021	44.4 (11,777)	44.3 (11,466)	49.4 (311)
*First evidence of shingles*			
During baseline, % (n)	–	–	1.4 (358)
*Shingles diagnosis in days (n = 358)*			
Median (IQR)	–	–	− 168 (− 269, − 84)
During follow-up, % (n)	–	–	1.0 (271)
*Shingles diagnosis in days (n = 271)*			
Median (IQR)	–	–	160 (64, 268)

SD, standard deviation; IQR, interquartile range. *Variables are described for the Medicare cohort only. **As part of the Data Use Agreement, cells are suppressed as *N* < 11 for patient deidentification purposes

**Table 2:** Censor reason, incidence rate (IR), and IR ratio (IRR) of outcomes up to 1-year post-surgery for the full cohort and based on diagnostic evidence of shingles among adults undergoing shoulder replacement

	Full cohort (*n* = 26,543)	No shingles cohort (*n* = 25,914)	Baseline or follow-up shingles cohort (*n* = 629)
*Primary outcome*			
Composite of 4 measures			
Censor reason, % (n)			
End of follow-up	80.3 (21,320)	80.2 (20,777)	86.3 (543)
Lost to follow-up	15.2 (4,040)	15.3 (3,976)	10.2 (64)
Event	4.5 (1,183)	4.5 (1,161)	3.5 (22)
Person time in years	23,510	22,929	582
IR (95% CI)	5.03 (4.75, 5.32)	5.06 (4.77, 5.35)	3.78 (2.20, 5.36)
IRR (95% CI)	–	Reference	0.75 (0.49, 1.14)
*Secondary outcomes*			
Surgical revision, % (n)	3.2 (860)	3.3 (841)	3.0 (19)
Irrigation/debridement, % (n)	0.3 (88)	0.3 (88)	0 (0)
Surgical removal, % (n)	0.4 (101)	< 0.5**	< 0.5**
Shoulder PJI, % (n)	1.3 (343)	< 1.4**	< 0.5**

CI, confidence interval; PJI, periprosthetic joint infection. **As part of the Data Use Agreement, cells are suppressed as *N* < 11 for patient deidentification purposes

**Table 3:** The association between shingles, treated in three different ways, and the composite of all four outcomes up to one year post-surgery among adults undergoing shoulder replacement (*n* = 26,543)

	Unadjusted	Model 1	Model 2*
	HR (95% CI)	HR (95% CI)	HR (95% CI)
*Treating shingles as baseline exposure as yes/no*			
No baseline shingles exposure	Reference	Reference	Reference
Baseline shingles exposure	1.13 (0.71, 1.80)	1.11 (0.70, 1.77)	1.10 (0.69, 1.76)
*Treating shingles as baseline exposure as quarterly exposure*			
No baseline shingles exposure	Reference	Reference	Reference
Baseline shingles exposure, 1st quarter closest to index surgery	0.91 (0.34, 2.43)	0.92 (0.34, 2.45)	0.90 (0.34, 2.39)
Baseline shingles exposure, 2nd quarter	0.90 (0.34, 2.4)	0.86 (0.32, 2.30)	0.86 (0.32, 2.30)
Baseline shingles exposure, 3rd quarter	1.44 (0.60, 3.46)	1.42 (0.59, 3.41)	1.41 (0.59, 3.40)
Baseline shingles exposure, 4th quarter furthest from index surgery	1.37 (0.57, 3.30)	1.35 (0.56, 3.24)	1.35 (0.56, 3.26)
*Treating shingles as time-varying exposure from baseline through follow-up as yes/no*			
No shingles exposure at baseline or follow-up	Reference	Reference	Reference
Shingles exposure at baseline or follow-up	0.92 (0.53, 1.60)	0.93 (0.54, 1.62)	0.94 (0.54, 1.63)

HR, hazard ratio; CI, confidence interval. Model one adjusted for age (as continuous), sex, and the Elixhauser Comorbidity Index. *As a sensitivity analysis, model two adjusted for the model one covariates + insurance type (Medicare, Commercial) and the year of surgery

## Data Availability

Data were retrieved from a 20% Medicare fee-for-service sample and the IBM^®^ MarketScan^®^ Commercial Database. Data for this study are available upon request from the corresponding author.
